# *Aedes aegypti* CLIPB9 activates prophenoloxidase-3 in the presence of CLIPA14 after fungal infection

**DOI:** 10.3389/fimmu.2022.927322

**Published:** 2022-07-28

**Authors:** Yannan Ji, Tengfei Lu, Zhen Zou, Yanhong Wang

**Affiliations:** ^1^ State Key Laboratory of Integrated Management of Pest Insects and Rodents, Institute of Zoology, Chinese Academy of Sciences, Beijing, China; ^2^ CAS Center for Excellence in Biotic Interactions, University of Chinese Academy of Sciences, Beijing, China

**Keywords:** immune melanization, prophenoloxidase, serine protease, serine protease homolog, antifungal response

## Abstract

Melanization is an integral part of the insect defense system and is often induced by pathogen invasion. Phenoloxidases (POs) are critical enzymes that catalyze melanin formation. PO3 is associated with the antifungal response of the mosquito, *Aedes aegypti*, but the molecular mechanism of the prophenoloxidase-3 (PPO3) activation is unclear. Here we report that PPO3 cleavage activation is mediated by a clip-domain serine protease, CLIPB9. We purified recombinant CLIPB9 and found that it cleaved PPO3 and increased PO activity in the hemolymph. We then identified CLIPA14 (a serine protease homolog) by co-immunoprecipitation using anti-CLIPB9 antibody. After being cleaved by CLIPB9, *Ae. aegypti* CLIPA14 acted as a cofactor for PPO3 activation. In addition, dsRNA co-silencing of *CLIPB9* and *CLIPA14* genes reduced melanization after infection with the entomopathogen, *Beauveria bassiana*, making the adult mosquitoes more sensitive to fungal infection. These results illustrate the roles of CLIPB9 and CLIPA14 in the PPO activation pathway and revealed the complexity of the upstream serine protease network controlling melanization.

## Introduction

Mosquitoes threaten public health by transmitting disease pathogens (1). *Aedes aegypti* transmits several devastating arboviruses, including dengue, yellow fever, Zika, and Chikungunya viruses (2). The entomopathogenic fungus, *Beauveria bassiana*, is an environmentally safe biological control agent that is harmless to non-target organisms (3). *B. bassiana* is a promising biopesticide, but successful fungal invasion of insect hosts involves overcoming the innate immune system.

Melanization is an essential component of the innate immunity in insects and crustaceans (4). Melanin synthesis is initiated with tyrosine oxidation by tyrosinase, which catalyzes the hydroxylation of tyrosine to 3, 4-dihydroxyphenylalanine (DOPA), followed by the subsequent oxidation of DOPA to dopaquinone. Dopaquinone is finally converted to melanin after a sequential range of reactions (5). The production of melanin in insects is catalyzed by phenoloxidase (PO), a key enzyme in the melanization cascade. Before melanization is activated, PO is present in its enzymatically inactive precursor form, prophenoloxidase (PPO). PPO is converted into its active form by a cascade of serine proteases (SPs) (6, 7). Once microorganisms or abnormal tissues are sensed by pattern recognition receptors in the circulatory system, a modular SP is activated, and downstream members of the SP cascade are sequentially cleaved within minutes (8–10).

Clip-domain serine proteases (CLIPs), which possess a large chymotrypsin-like fold and one or two clip domains, are important in the SP cascade (11). Mosquito CLIPs can be divided into five branches: A–E (11–13). The carboxyl-terminal domain of CLIPB–Ds contain the catalytic triad His-Asp-Ser. There are three SP cascades for PPO activation in the biochemical model *Manduca sexta*. Microbial recognition causes autoactivation of hemolymph protease-14 precursor (proHP14), and activated HP14 cleaves proHP21, a trypsin-like SP that converts proPAP2/3 to PAP2/3 (14–16). In another cascade, proHP1 somehow activates proHP6 (17, 18), and HP6 cleaves proPAP1 for PPO activation and proHP8 for proSpätzle-1 activation (19, 20). The last cascade is activated in wandering larvae and pupae, which is composed of HP14, HP2, and PAP2 (21). Both HP21 and PAP3 activate proHP5, which cleaves proHP6, thus connecting the first two SP cascades (22). Exploration of SP cascades can increase understanding of the molecular mechanism of melanization.

CLIPAs and CLIPEs are serine protease homologs (SPHs) that contain an amino-terminal clip domain, but the catalytic residues in the protease-like domain are replaced by other residues (23). Some SPHs serve as efficient cofactors of PAPs to enhance PPO activation in lepidopteran and coleopteran insects, such as SPH1/SPH2 in *M. sexta* (15, 24), cSPH11/cSPH50 in *Helicoverpa armigera* (25) and PPAF-II in *Holotrichia diomphalia* (26). Moreover, some SPHs can exert their effects upstream of the melanization cascade by regulating the activation of CLIP-SPs. For example, CLIPA8 and CLIPA28 are located upstream of CLIPC9 to regulate the melanization immune response in *Anopheles gambiae* (27).

There are two different melanization activation pathways in *Ae. aegypti*, which are carried out by different SPs and regulated by specific serpins. Tissue melanization is usually associated with the destruction of host tissue and is controlled by serpin-2, CLIPB8, and IMP-1. Immune melanization is mediated by IMP-1, IMP-2, and serpin-1, which activate hemolymph PPOs against malaria parasites (28). Our previous study showed that PPO3 can be cleaved by *Ostrinia furnacalis* SP105 (*Of*SP105) *in vitro* and PO3 plays a key role in antifungal responses (29). However, the detailed mechanism of PPO activation in *Ae. aegypti* is unknown.

In this study, we identified CLIPs involved in the PPO activation in *Ae. aegypti* using a biochemical approach. We found that CLIPB9 directly cleaved PPO3 to become PO3. CLIPA14, an SPH, was identified as a cofactor of CLIPB9 to enhance PPO activation. Silencing of *CLIPB9* and *CLIPA14* reduced the melanization caused by *B. bassiana* infection.

## Materials and methods

### Experimental animals, microbial culture, immune challenge, and hemolymph collection

The wild-type strain (Liverpool) of *Ae. aegypti* was maintained in our laboratory (30). Adult mosquitoes were fed on water and 10% sucrose solution for maintenance. Chickens provided blood meals for the adult mosquitoes. All procedures using vertebrates were approved by the Animal Care and Use Committee of the Institute of Zoology, Chinese Academy of Sciences. *B. bassiana* (ARSEF2860) used for the immune challenge was cultured on Potato Dextrose Agar plates at 26 °C. Mosquito infection was performed by the abdomen needle pricking method (microbial solution concentration: *B. bassiana*, 1 × 10^8^ conidia/ml). Fresh hemolymph was collected from female mosquitoes as described (28), and serum was obtained after 5,000 × *g* centrifugation for 5 min at 4 °C.

### RNA isolation and quantitative real-time PCR

Total RNA was extracted from homogenized adult female mosquitoes using TRIzol (Invitrogen). The cDNA was then reverse transcribed using a PrimeScript™ RT Reagent Kit with gDNA Eraser (TaKaRa). SuperReal PreMix Plus SYBR Green (Tiangen) combined with Applied Biosystems Step One Plus Real-Time PCR System (Thermo Fisher Scientific) was used for quantitative real-time PCR (qRT-PCR) reaction. We used the 2^−ΔΔCt^ method to determine the relative expression levels of the corresponding genes. The primers were designed by software Primer 5, and rps7 served as an internal control. The primers are listed in **Table S1**. All values are expressed as mean ± standard errors of the means (SEM). We used Student’s t test to calculate the differences between samples (GraphPad).

### Expression and purification of proCLIPB9_Xa_, proCLIPA14_Xa_, and PPO3 proteins

To produce the near full-length of CLIPB9 and CLIPA14 transcripts for eukaryotic expression, cDNA was amplified by PCR using specific primers (**Table S1**). The PCR product was cloned into the pMT-BiP/V5-HisA vector (Invitrogen). Putative cleavage sites of proCLIPB9 and proCLIPA14 are IGMR^136^ and LGFR^163^. The four residues of the hypothetical cleavage site were replaced with the IEGR tetrapeptide recognized by Factor Xa (New England Biolabs) by the overlap extension PCR method, and the recombinant plasmids of proCLIPB9_Xa_ and proCLIPA14_Xa_ were obtained. The recombinant plasmid and pCoHygro hygromycin selection vector (Invitrogen) were co-transfected into *Drosophila* S2 cells cultured in SFX medium (HyClone) at 28 °C, and stable cell lines were selected. After induction with 500 μM copper sulfate, the supernatants were collected for recombinant protein purification. Then, protein was purified using Ni-NTA agarose. The concentration of the purified protein was estimated by bicinchoninic acid (BCA) assay and analyzed using SDS-PAGE followed by immunoblotting. As for recombinant PPO3, it was purified with Ni-NTA agarose as described previously (31). The purified products were stored at −80 °C until use.

### Assay for serine protease hydrolase activity and PO activity

In order to examine whether the recombinant proteins proCLIPB9_Xa_ and proCLIPA14_Xa_ can be activated by Factor Xa, 100 ng purified recombinant proCLIPB9_Xa_ or proCLIPA14_Xa_ and Factor Xa were incubated in the buffer (2 mM CaCl_2_, 0.1 M NaCl, 20 mM Tris-HCl, pH8.0) at 37 °C. The amidase activity of the samples was measured using 50 μM acetyl-Ile-Glu-Ala-Arg-*p*-nitroanilide (IEAR) as the substrate. Enzyme activity was measured by absorbance at 405 nm, and one unit of enzyme activity was defined as an increase in absorbance of 0.001/min.

The mixture, which included activated SPs and fresh hemolymph or purified PPO3, was incubated for 10 minutes before immunoblotting. PO activity was measured according to previous studies (22). PO activity was determined using dopamine solution (2 mM dopamine dissolved in 50 mM sodium phosphate solution, pH 6.5). Absorbance was measured at 490 nm, and PO activity (one unit) was defined as the amount of enzyme that changed absorbance by 0.001/min.

### Preparation of polyclonal antibodies

Full-length of CLIPB9 or CLIPA14 was amplified by PCR using specific primers (**Table S1**) and cloned into the prokaryotic expression plasmid pET-28a. Recombinant protein was expressed in *Escherichia coli* strain BL21 (DE3) cells and purified with Ni-NTA agarose. His-tagged target proteins bound to Ni-NTA resin were eluted with buffer (300 mM NaCl, 50 mM sodium phosphate buffer, 200 mM imidazole) containing 8 M urea. The recombinant proteins were sent to Beijing Protein Innovation for the preparation of antibodies.

### Immunoblotting analysis

For immunoblotting analysis, the proteins were separated on 4-20% Tris-Glycine gels (RSBM) and transferred electrophoretically onto PVDF membranes (Millipore). The membranes were blocked with StartingBlock™ T20 (TBS) Blocking Buffer (Thermo Fisher Scientific) at room temperature and then incubated with primary antibodies at 4 °C overnight. The following antibodies were used: anti-V5 antibody (Invitrogen), anti-CLIPB9 and -CLIPA14 antibody (prepared by Beijing Protein Innovation), and anti-PPO3 antibody (29).

### Co-immunoprecipitation

Co-immunoprecipitation assays were performed as described (32). Briefly, 24 h *B. bassiana*-infected hemolymph and PBS-treated hemolymph were collected. Samples were incubated with 50 μl Protein A-Sepharose (Roche) to remove nonspecific binding. After centrifugation, specific antibodies were added to the supernatant and incubated at 4 °C for 1 h. Subsequently, 50 μl of Protein A was added to each sample, which was then shaken at 4 °C for at least 3 h, or overnight. The Protein A was separated by centrifugation. The target and its associated proteins were washed at least four times with NP-40 lysis buffer as described in the kit and then disrupted and resolved by SDS-PAGE.

### Sample preparation and data analysis for mass spectrometry

Equal amounts of protein samples were separated on 4–20% Tris-Glycine gels and visualized by Coomassie Brilliant Blue (CBB) stain. Visible bands were excised from the gel and cut into 1-mm^3^ pieces. The pieces were subjected to in-gel reduction with dithiothreitol, alkylation with iodoacetamide, and then digested with mass spectrometry grade trypsin (Promega). The tryptic peptides were extracted with 5% (v/v) trifluoroacetic acid in 50% (v/v) acetonitrile, concentrated by drying under vacuum conditions, and dissolved in mobile phase A buffer (0.1% formic acid). The supernatants were then analyzed using liquid chromatography-tandem mass spectrometry (LC-MS/MS). Peptide and protein identification and peak area calculations were performed against the *Ae. aegypti* proteome database using Proteome Discoverer 2.4.1.15 (Thermo Fisher Scientific).

### Synthesis and micro-injection of dsRNAs

PCR primers were designed to contain forward and reverse primers to amplify DNA fragments of CLIPB9 (563 bp) and CLIPA14 (534 bp), which contained both sense and antisense T7 promoter sequences. The oligonucleotide primers are listed in **Table S1**. The PCR product was purified using the QIAquick PCR Purification Kit (QIAGEN) and dsRNA was synthesized with the T7 RiboMAX Express RNAi Systems kit (Promega) according to manufacturer protocol. The enhanced green fluorescent protein (EGFP) dsRNA was used as a control. Adult female mosquitoes, collected within 24 h of emergence, were injected with corresponding dsRNA (1.2 μg per 207 nl) using a Nanoliter 2000 syringe (World Precision Instruments) as describe (33). The efficiency of dsRNA-mediated gene silencing was determined by collecting eight mosquitoes at 4 d post-injection.

### Survival rate analysis

Mosquitos were infected with *B. bassiana* conidia by needle pricking method after being treated with dsRNA for 48 h. The mosquitoes were kept in separate spaces, and each test group consisted of 30 mosquitoes.

### Statistical analysis

Data were analyzed using GraphPad Prism software. Before statistical analysis, the independence, normality, and homogeneity of variances of experimental data were tested. All experiments in this study were repeated at least three independent times. Survival rate analysis was executed using Kaplan-Meier survival curves, and comparisons were done using the log-rank/Mantel-Cox test. Date were displayed as means ± SEM. Differences were considered to be statistically significant when the calculated P value was less than 0.05.

### Gene accession numbers

All sequences supporting the findings of this study are available in the NCBI Protein Database under the following accession numbers: CLIPB9 (XP_021711027.1), CLIPA14 (EAT46191.1), PPO3 (EAT36126.1).

## Results

### *B. bassiana* induces melanin synthesis in *Ae. aegypti*

**Figure 1:**
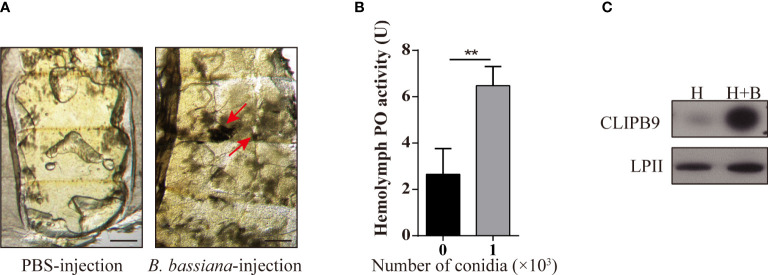
*B. bassiana* induced melanin synthesis is mediated by *Ae. aegypti* CLIPB9. **(A)** Mosquitoes appeared melanization after infected with *B. bassiana*. The melanotic masses were found in mosquitoes infected with *B. bassiana* but not in control PBS treatment. Abdominal images were observed under an OLYMPUS SZX16 microscope. The red arrows indicate the melanotic masses. Scale bars, 200 μm. **(B)** PPO in hemolymph was activated by *B. bassiana* conidia. PO activity was detected after 10-fold dilution of fresh hemolymph. The hemolymph with the addition of 1 × 10^3^ conidia was significantly activated compared to the hemolymph without the addition of conidia. The bars represent the mean ± SEM (n = 3). **P < 0.01. **(C)** Immunoblotting analysis of CLIPB9 expression in hemolymph after *B. bassiana* infection. H, mosquito hemolymph was uninfected. H + B, mosquito hemolymph infected with *B. bassiana*. Anti-LPII antibody was used as the loading control.

Melanization plays an important role in mosquito resistance to fungal infection (34). To investigate its effect on humoral immunity of *Ae. aegypti* in response to *B. bassiana*, we used a conidia suspension (1 × 10^8^ conidia/ml) of *B. bassiana* to infect adult female mosquitoes with needle inoculation. After 24 h, the melanotic masses appeared on the abdomen of the mosquitoes in the experimental groups, whereas no obvious melanotic masses were observed in the control groups (**Figure 1A**). The key biosynthetic enzyme in insects for the production of melanotic masses is PO (35). In order to detect the activity of PO in hemolymph, 1 × 10^3^ conidia were added to the fresh mosquito hemolymph which reacted in the dopamine solution. We found that the PO activity in the hemolymph after adding *B. bassiana* conidia was approximately 2.5-fold higher than that of the control hemolymph (2.65 ± 1.23 U vs. 6.48 ± 0.84 U, P < 0.01) (**Figure 1B**). This shows that *B. bassiana* can induce melanin synthesis and increase hemolymph PO activity.

The protease domain of the CLIPB subfamily can cleave PPO to PO (36). *Ae. aegypti* CLIPB9 (IMP-1), *Holotrichia* PPAFI, *Drosophila* MP2, and *M. sexts* PAP-1 belong to the same group in the phylogenetic analysis (28). Enrichment analysis of the published transcriptome data (31) showed that CLIPB9 was significantly up-regulated after *B. bassiana* infection from the systematic cluster analysis (**Figure S1**). Likewise, Northern blot analysis detected substantial enrichment of CLIPB9 (IMP-1) after microbial and fungal infection (28). The protein level of CLIPB9 was also significantly induced in the hemolymph after infection with *B. bassiana* for 24 h (**Figure 1C**).

### Structural features, purification, and activation of recombinant proCLIPB9

**Figure 2:**
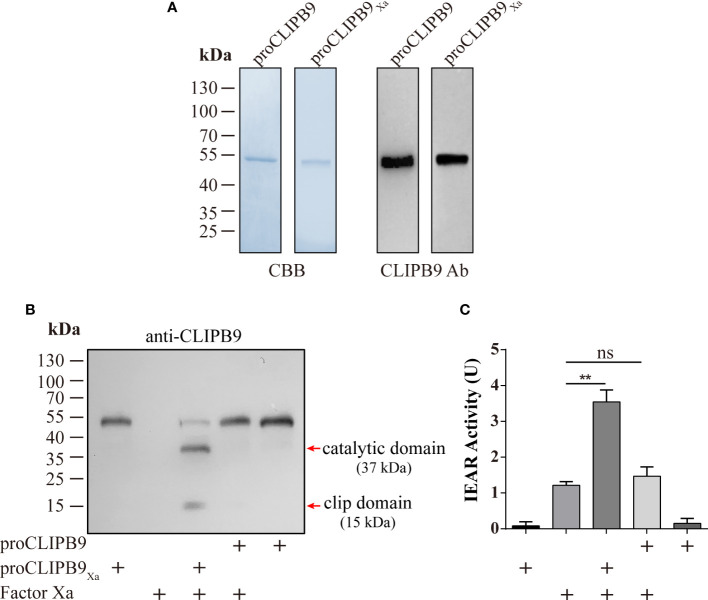
Purification and activation of recombinant proCLIPB9 and proCLIPB9_Xa_. **(A)** Purified recombinant proCLIPB9 and proCLIPB9_Xa_ were analyzed by SDS-PAGE and immunoblotting. Left, CBB staining; Right, immunoblotting assays using anti-CLIPB9 antibody (CLIPB9 Ab). **(B)** The cleavage of purified proCLIPB9_Xa_ by Factor Xa was detected by immunoblotting. After incubation of Factor Xa with proCLIPB9 (100 ng) or proCLIPB9_Xa_ (100 ng), immunoblotting was performed using anti-CLIPB9 antibody. **(C)** Amidase activity assay of CLIPB9_Xa_. The catalytic activity of CLIPB9_Xa_ cleaved by Factor Xa was detected using IEAR*p*Na as a substrate. The bars represent the mean ± SEM (n = 3). **P < 0.01; ns, P > 0.05.

CLIPB9 was considered to mediate the melanization response in *Ae. aegypti* and confirmation required biochemical evidence. The proCLIPB9 cDNA sequence encodes a signal peptide and a clip domain, followed by a carboxyl-terminal protease domain that contains a catalytic triad of His^182^, Asp^246^, and Ser^337^. To investigate the exact function of CLIPB9 in the melanization response of *Ae. aegypti*, we generated recombinant CLIPB9 *in vitro* using *Drosophila* S2 cells. Since the endogenous activating enzyme of CLIPB9 is unknown, we expressed and purified the zymogen form (proCLIPB9) and activation site modified form (proCLIPB9_Xa_) of CLIPB9. In proCLIPB9_Xa_, the predicted activation site IGMR^136^ was replaced with the IEGR^136^ tetrapeptide recognized by Factor Xa. The purified proCLIPB9 and proCLIPB9_Xa_ migrated as a 50 kDa band on the CBB-stained gel and could be recognized by anti-CLIPB9 antibody (**Figure 2A**).

The potential activation cleavage site of proCLIPB9 is between R^136^ and I^137^. After the purified proCLIPB9_Xa_ (100 ng) was incubated with Factor Xa, intensity of the zymogen band at 50 kDa decreased and was replaced by two bands at 37 and 15 kDa (**Figure 2B**), corresponding to the catalytic and clip domains. After incubation with Factor Xa, the proCLIPB9 band was not cleaved (**Figure 2B**). Furthermore, the activity of CLIPB9 was indicated by hydrolysis of the IEAR*p*NA substrate. Although Factor Xa showed some activity with this substrate, a significant increase in activity was observed after incubation with proCLIPB9_Xa_. No significant changes were observed after incubation with proCLIPB9. Since proCLIPB9_Xa_ or proCLIPB9 alone had no amidase activity (**Figure 2C**), the recombinant proCLIPB9_Xa_ cleaved by Factor Xa was an active IEARase.

### CLIPB9_Xa_ can cleave and activate recombinant and native PPO3 of *Ae. aegypti*

**Figure 3:**
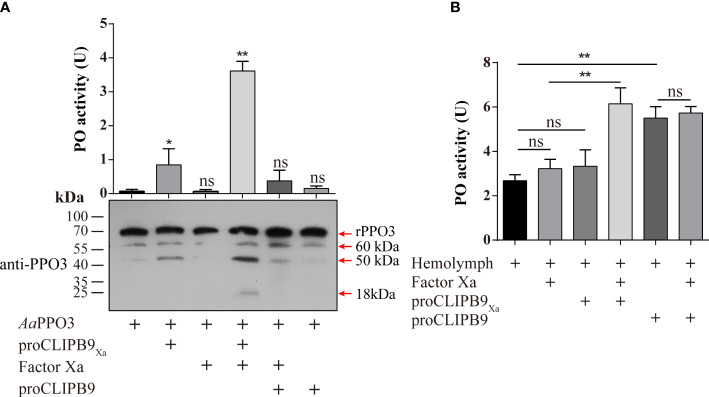
Cleavage activation of *Ae. aegypti* PPO3 by Factor Xa-treated proCLIPB9_Xa._ **(A)** PPO3 was activated by CLIPB9. Recombinant PPO3 was incubated with buffer, Factor Xa, proCLIPB9, proCLIPB9_Xa_, or Factor Xa and proCLIPB9/proCLIPB9_Xa_. The mixtures containing 300 ng PPO3 were used in the PO activity assay (upper panel), and the mixtures containing 100 ng PPO3 were analyzed by immunoblotting using PPO3 antibodies (bottom panel). **(B)** CLIPB9 activates PPO in hemolymph. The 1:10 diluted hemolymph samples were incubated with proCLIPB9 or Factor Xa-treated proCLIPB9Xa. PO activities were measured using dopamine as a substrate and plotted as mean ± SEM (n = 3). **P < 0.01; *P < 0.05; ns, P > 0.05.

The previous study indicated that PPO3 was cleaved in the hemolymph of fungal challenged mosquitoes and this response could be inhibited by CLSP2 (31). Recombinant PPO3 (300 ng) was added to the different samples and detected by immunoblotting using anti-PPO3 antibody. As expected, rPPO3 was efficiently cleaved into three bands of 60, 50, and 18 kDa by CLIPB9_Xa_ in the presence of Factor Xa (**Figure 3A**, bottom panel), and high PO activity (3.615 U) was observed (**Figure 3A**, upper panel). There was little PO activity of rPPO3, however, cleavage of rPPO3 by some partially activated CLIPB9 is likely. This could explain the weak PO activity in the figure (**Figure 3A**, upper panel), but it does not imply that the proenzyme activates rPPO3.

To examine the role of CLIPB9 in the hemolymph melanization *in vitro*, proCLIPB9 or Factor Xa-treated proCLIPB9_Xa_ was mixed with hemolymph at 37 °C for 20 min. We found that the PO activity of hemolymph after adding activated CLIPB9_Xa_ and proCLIPB9 was about 1.9-fold (3.22 ± 0.43 U vs. 6.14 ± 0.82 U, P < 0.01) and 2.0-fold (2.68 ± 0.30 U vs. 5.51 ± 0.59 U, P < 0.01) higher than that of the controls (**Figure 3B**), indicating that CLIPB9 acts as the PPO activation enzyme.

### Identification CLIPB9 and its potential interaction protein CLIPA14

**Figure 4:**
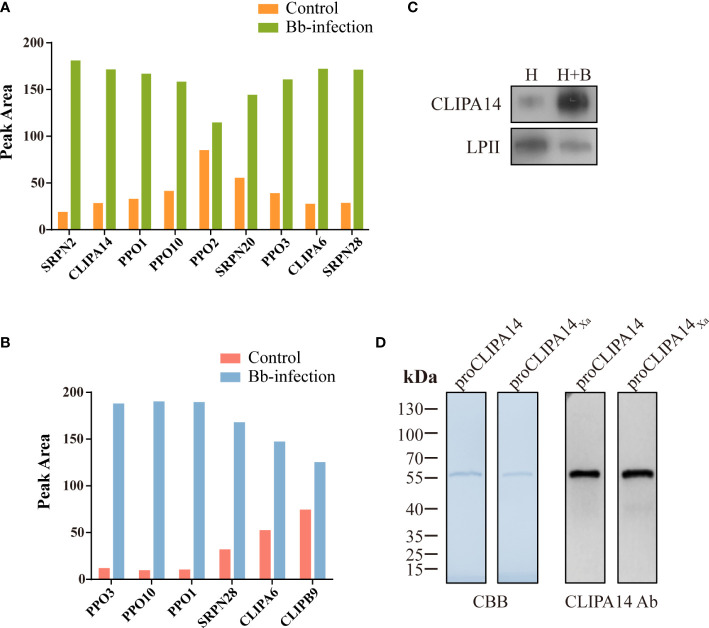
A potential interacting protein of CLIPB9. Proteomic analysis of CLIPB9 **(A)** and CLIPA14 **(B)** associated proteins. Co-IP experiments were performed on Bb-infected and control cell-free hemolymph using anti-CLIPB9 or anti-CLIPA14 antibody. Proteins eluted from the immunoaffinity columns were treated with trypsin for LC-MS/MS analysis. Peak processing was performed using Proteome Discoverer 2.4.1.15 (Thermo Fisher Scientific) software. **(C)** Immunoblotting analysis of CLIPA14 expression in hemolymph after *B. bassiana* infection. H, mosquito hemolymph was uninfected. H + B, mosquito hemolymph infected with *B. bassiana*. Anti-LPII antibody was used as the loading control. **(D)** Purified recombinant proCLIPA14 and proCLIPA14_Xa_ analyzed by SDS-PAGE and immunoblotting. Left, CBB staining. Right, immunoblotting using anti-CLIPA14 antibody.

To identify proteins that interact with CLIPB9, we captured CLIPB9 and its associated proteins in fungal-infected and uninfected cell-free hemolymph using a Co-IP coupled quantitative proteomic approach. CLIPB9 related protein complex was isolated from hemolymph samples using anti-CLIPB9 antibody. After trypsin digestion, the peptide fragments were studied by LC-MS/MS for label-free quantitative analysis. A total of 48 proteins (**Table S2**) were identified from the complex, among which a series of melanization related proteins such as PPOs (PPO1−3, and 10) and serpins (SRPN2, 20, and 28) significantly increased in the hemolymph after infection (**Figure 4A**). This result showed that PPO3 formed a complex with CLIPB9, which further supported that CLIPB9 participates in PPO3-mediated immune melanization. In addition, the complex formed by PPO3 and CLIPB9 also contains several other PPOs and serpins, suggesting that these molecules may also be involved in immune melanization.

Interestingly, two SPHs (CLIPA14 and CLIPA6) identified in the CLIPB9 pulldown were still up-regulated (**Figure 4A**). We speculate that CLIPA14 and CLIPA6 may be potential cofactors for PPO activation. In the present study, we focused on CLIPA14 first. We used anti-CLIPA14 antibody to Co-IP with *B. bassiana* infected and uninfected cell-free hemolymph and identified a total of 17 proteins (**Table S3**). Similarly, CLIPB9, PPO3, CLIPA6, and other proteins associated with melanization were also significantly up-regulated in the hemolymph infection with *B. bassiana* (**Figure 4B**), indicating that CLIPA14, CLIPA6, and CLIPB9 may form a complex with PPO3. The anti-CLIPB9 antibody can’t recognize the CLIPA14 protein and vice versa (**Figure S2A**). To verify the interaction between CLIPB9 and CLIPA14, we conducted reciprocal Co-IP immunoblotting experiments (**Figure S2B**). Results showed that when CLIPB9 protein was immunoprecipitated from hemolymph using the anti-CLIPB9 antibody, CLIPA14 was detected in the immunocomplex using the anti-CLIPA14 antibody and vice versa. CLIPB9 and CLIPA14 can be identified in hemolymph using the anti-CLIPB9 and anti-CLIPA14 antibodies, respectively.

### Enhancement of PPO activation in the presence of CLIPA14

In order to study the role of CLIPA14 in PPO activation, we first examined the protein sequence of CLIPA14. The proCLIPA14 consists of 428 amino acids, including a signal peptide, a clip domain followed by a carboxyl-terminal protease-like domain. CLIPA14 contains 16 conserved Cys residues and its putative proteolytic activation site is located after LGFR^163^. Subsequently, the presence of CLIPA14 in hemolymph was detected by its antibody. Level of the proCLIPA14 was significantly up-regulated after fungal infection (**Figure 4C**), consistent with the proteomic result (**Figure 4A**). We expressed the recombinant proCLIPA14 and its modified forms (proCLIPA14_Xa_) in *Drosophila* S2 cells and purified them on a Ni-NTA column. CBB-stained SDS-PAGE gel of proCLIPA14 and proCLIPA14_Xa_ showed a single 55 kDa band recognized by anti-CLIPA14 antibody (**Figure 4D**).

**Figure 5:**
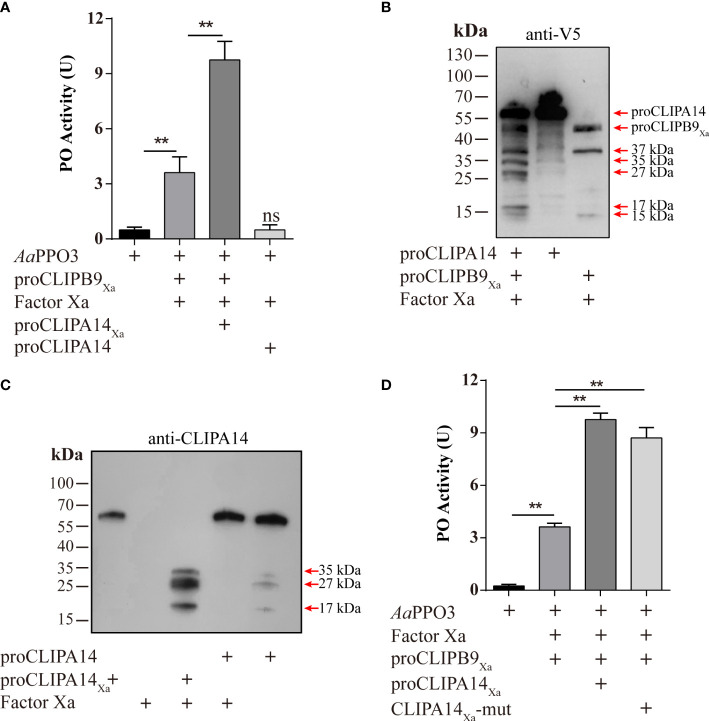
*Ae. aegypti* CLIPA14 treatment significantly increased the PO activity. **(A)** Enhancement of PPO3 activation by CLIPA14. proCLIPB9_Xa_ or its mixture with proCLIPA14_Xa_ was treated with Factor Xa and then incubated with 300 ng PPO3 prior to PO activity measurement. **(B)** The cleavage of proCLIPA14 by activated CLIPB9_Xa_ was detected by immunoblotting. Factor Xa-treated CLIPB9_Xa_ was incubated with proCLIPA14 (100 ng) and the mixture and controls were analyzed by immunoblotting using anti-V5 antibody. **(C)** The cleavage of purified proCLIPA14_Xa_ by Factor Xa was detected by immunoblotting. After incubation of Factor Xa with proCLIPA14 (100 ng) or proCLIPA14_Xa_ (100 ng), the mixture was immunoblotted with anti-CLIPA14 antibody. **(D)** The CLIPA14_Xa_-mutant can also act as a cofactor to increase PO activity. ProCLIPB9_Xa_, proCLIPA14_Xa_, and proCLIPA14_Xa_-mutant were activated by Factor Xa and then incubated with PPO3. Using dopamine as the substrate to measure PO activity. CLIPA14_Xa_-mut, CLIPA14_Xa_-mutant. The bars represent the mean ± SEM (n = 3). **P < 0.01; ns, P > 0.05.

After Factor Xa-treated proCLIPA14_Xa_ (100 ng) was incubated with rPPO3 or a mixture of rPPO3 and Factor Xa-treated proCLIPB9_Xa_, a PO activity assay showed that addition of CLIPB9_Xa_ and CLIPA14_Xa_ significantly increased the PO activity by 2.7-fold compared with addition of CLIPB9_Xa_ only (3.62 ± 0.96 U vs. 9.76 ± 1.15 U, P < 0.01) (**Figure 5A**). Likewise, proCLIPA14 was added to the hemolymph to examine the role of CLIPA14 in the hemolymph melanization *in vitro*. We found that the PO activity of hemolymph after adding proCLIPA14 was approximately 1.95-fold than that of the control (2.78 ± 0.42 U vs. 5.43 ± 0.76 U, P < 0.01) (**Figure S3**).

In *M. sexta*, proSPH1 and 2 were cleaved by the PAPs before executing their function (15). To test whether proCLIPA14 could be cleaved by PAP before functioning in *Ae. aegypti*, the activated CLIPB9_Xa_ was incubated with proCLIPA14 and then analyzed by immunoblotting using anti-V5 antibody. As expected, 35, 27, and 17 kDa bands were detected after the treatment (**Figure 5B**), consistent with activated CLIPA14_Xa_ which was cleaved by Factor Xa (**Figure 5C**).

In *M. sexta*, the significant increase of PO activity was dependent on the co-presence of SPH1 and SPH2 (15). In the *M. sexta* SPH1 and SPH2 sequences, the conserved motifs of GDSGGP were replaced by GDGGSP and GDGGAP, respectively. In the sequence of CLIPA14, we mutated GDGGSP to GDGGAP, resulting in a CLIPA14Xa mutant, to test whether it still produces cofactor activity. PO activity assays were performed after incubation with Factor Xa-treated proCLIPB9Xa and PPO3. We found that PO activity was significantly enhanced in the inclusion of CLIPA14 mutants compared to controls (**Figure 5D**). This suggests that CLIPA14 can replace the co-existence of SPH1 and SPH2 to exert cofactor activity.

### Effect of *Ae. aegypti* CLIPB9 and CLIPA14 on *B. bassiana* infection

**Figure 6:**
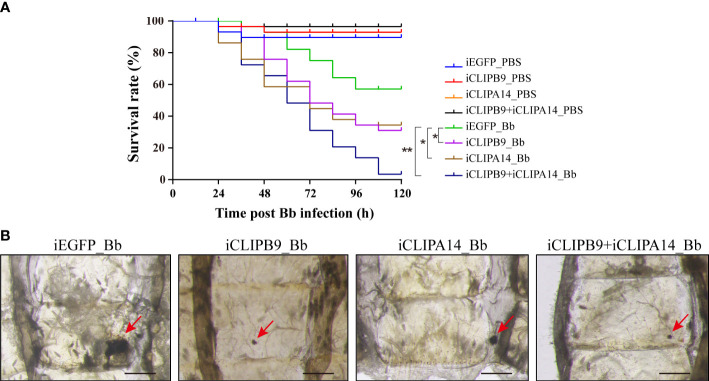
The roles of CLIPB9 and CLIPA14 in mosquito antifungal defense. **(A)** Effects of silencing of *CLIPB9* and *CLIPA14* on the survival rate of mosquitoes after *B. bassiana* (Bb) infection. Depletion of CLIPB9 or CLIPA14 reduced mosquito resistance to *B. bassiana* compared to the control group (iEGFP_Bb), and concomitant depletion of CLIPB9 and CLIPA14 further reduced the resistance of mosquitoes to *B. bassiana*. Three biological repeats were conducted. The significance between different survival curves was compared using the log-rank (Mantel-Cox) test. *, P < 0.05; **P < 0.01. **(B)** Effects of silencing *CLIPB9* and *CLIPA14* on melanization in mosquitoes after *B. bassiana* infection. Compared to the control group (iEGFP_Bb), the melanotic masses on the abdomen of mosquitoes were reduced by varying degrees in the CLIPB9-depleted group, the CLIPA14-depleted group, and the co-silenced group. Abdominal images were observed under an OLYMPUS SZX16 microscope. The red arrows indicate the melanotic masses. Scale bars, 200 μm.

To support the connection between CLIPB9 and CLIPA14, we investigated their ability to block antifungal infection by RNA interference (RNAi). We conducted double-stranded RNA (dsRNA) mediated knockdown by injecting dsRNA of *CLIPB9* or *CLIPA14* into mosquitoes within the first 24 h after eclosion. The specific knockdown of CLIPB9 and CLIPA14 was confirmed by immunoblotting and qRT-PCR (**Figure S4**). The survival rate in the mosquitoes with CLIPB9 RNAi depletion (iCLIPB9) was reduced by 26.7% compared to iEGFP mosquitoes (**Figure 6A**). The survival rate was also reduced by 23.3% in iCLIPA14 mosquitoes. In addition, the survival rate of mosquitoes with co-knockdown of iCLIPB9 and iCLIPA14 was reduced to 6.6% (**Figure 6A**).

Next, we studied their contribution to the mosquito melanization after fungal infection. Results showed that iEGFP mosquitoes infected with *B. bassiana* obviously appeared melanotic masses, whereas iCLIPB9 mosquitoes detected only a small melanotic spot (**Figure 6B**), indicating that the melanization mediated by CLIPB9 was weakened. In addition, the melanotic masses in iCLIPA14 mosquitoes infected with *B. bassiana* also appeared to be reduced (**Figure 6B**), suggesting that CLIPA14 is involved in the formation of melanization. This is consistent with the decreased survival rate after dsCLIPA14 treatment (**Figure 6A**). However, the size of melanotic spot in co-silencing of *CLIPB9* and *CLIPA14* is smaller than that in silencing of *CLIPA14* alone, but looks similar to that in silencing of *CLIPB9* alone (**Figure 6B**), indicating that co-silencing of *CLIPB9* and *CLIPA14* can’t block the immune melanization completely. In conclusion, our experiments demonstrated that CLIPA14 acts as a cofactor of CLIPB9 in the antifungal immune melanization.

## Discussion

We identified biochemical function of CLIPB9, which directly cleaves and activates *Aa*PPO3 *in vivo* and *in vitro*. CLIPA14 was also identified as an interacting protein of CLIPB9 by Co-IP, and further studies demonstrated that CLIPA14 acts as a cofactor of CLIPB9 to enhance the activity of PO. dsRNA silencing of CLIPB9 and CLIPA14 genes reduced melanotic masses in mosquitoes after *B. bassiana* infection. Finally, our studies showed that CLIPB9 and CLIPA14 are both involved in the antifungal immune melanization response.

The PPO activation pathway is composed with multiple CLIPs and serpins. Currently, PPO activating enzymes that directly cleave PPO belong to the CLIPB subfamily. For instance, cSP6/cSP8 in *H. armigera* (25, 32), PAP1/PAP2/PAP3 in *M. sexta* (15, 24, 37), PPAE in *B. mori* (38), SP13/SP105 in *O. furnacalis* (39, 40), and MP2 in *D. melanogaster* (41). The recombinant *An. gambiae* CLIPB9 and CLIPB10 can cleave the purified *M. sexta* PPO protein *in vitro* (42, 43). In the present study, we successfully obtained the soluble proCLIPB9 (**Figure 2A**) of *Ae. aegypti*, which was found to activate native and recombinant *Ae. aegypti* PPO3 (**Figure 3**). In addition, dsRNA silencing of CLIPB9 reduced melanization after *B. bassiana* infection and made adult mosquitoes susceptible to fungal infection (**Figure 6**).

The mechanism by which the terminal CLIPs activate PPO in the presence of cofactors has been characterized in insects (44). In *M. sexta*, PPO exhibited low PO activity after direct cleavage by PAP1, PAP2, and PAP3, but the activation was significantly enhanced in the presence of SPH1 and SPH2 (15, 23, 37). In *H. armigera*, the PPO activation pattern was analogous to that of *M. sexta*, and the activity of SP6 to activate PPO significantly increased when cSPH11 and cSPH50 were present (25). In this reaction, SPHs need to be cleaved by PPO activating enzymes in order to exert their effects. In *Ae. aegypti*, we used Co-IP to discover the cofactors of PPO activating enzymes. The hemolymph of mosquitoes infected with *B. bassiana* was subjected to Co-IP analysis with the anti-CLIPB9 antibody, and the immunoprecipitated proteins were identified and quantitatively analyzed by LC-MS/MS (**Figure 4A**). The identified CLIPA14 and CLIPA6 were notable and we found them to be orthologous to *M. sexta* SPHs. In this study, we only focused on CLIPA14. PO activity was significantly increased in the presence of cofactor CLIPA14 (**Figure 5A**). The cofactor CLIPA14 was also cleaved by CLIPB9 to produce three cleavage bands (**Figure 5B**). However, it is still unknown which cleavage product is the active cofactor.

Interestingly, the co-presence of two SPHs is necessary for the cofactor activity in lepidopteran insects (25, 45), whereas our study showed a single SPH can achieve the same effect in dipteran *Ae. aegypti*. In SPs, three highly conserved catalytic residues including histidine, aspartic acid, and serine (46) are located in a groove, known as the catalytic pocket, on the surface of the protein (47). The catalytic triad of His, Asp, and Ser is the most conserved feature in the other hydrolase families (48, 49). Analysis of the CLIPA14 sequence revealed that only Ser is missing among the three catalytic residues. Given their overall structural similarity and catalytic pocket sequence similarity, we speculate that CLIPA14 cofactor activity is more complete compared to *M. sexta* SPH1/SPH2 and *H. armigera* cSPH11/cSPH50. This may explain why CLIPA14 itself can exert cofactor activity (**Figure 5A**).

In *An. gambiae*, SPHs are regulators of TEP1 mediated immune response against malaria parasites and other microbial infections (50). Among them, CLIPA8, SPCLIP1, and CLIPA28 act as positive regulators (34, 51, 52), while CLIPA2 and CLIPA14 act as negative regulators of upstream CLIPs (53, 54). The exact function of these SPHs is unclear, but their RNAi phenotype suggests multiple reactions regulating the melanization. All the SPHs appear to play important roles in the melanization reaction. They either act as cofactors to increase the activity of PO or regulate the activation of CLIPs upstream of the melanization cascade. We found that CLIPA14 is involved in PO activation as a cofactor downstream of the SPs cascade (**Figure 5A**). Furthermore, the size of melanotic spot in the co-silencing of *CLIPB9* and *CLIPA14* looks similar to that in the silencing of *CLIPB9* alone (**Figure 6B**), suggesting that the co-silencing of *CLIPB9* and *CLIPA14* can’t block the immune melanization completely. One of the most likely reasons is that CLIPB9 can directly cleave PPO to affect the occurrence of melanization. Additionally, *Ae. aegypti* has 10 PPO genes and 96 CLIPs (55), and the expansion of the mosquito melanization cascade gene suggests the highly complexity of this system in mosquitoes (13). Melanization was not completely abolished in the co-silencing of *CLIPB9* and *CLIPA14*, suggesting that other serine protease cascade pathways may affect fungal-induced melanization. In conclusion, we propose a new model of the *Ae. aegypti* immune melanization pathway in response to fungal infection (**Figure S5**). At the end of the protease cascade, CLIPB9 acts as a PPO activating enzyme to cleave and activate *Aa*PPO3 in the presence of the cofactor CLIPA14, resulting in melanization in response to *B. bassiana* infection. In addition, Serpin-1, Serpin-2, CLSP2 and CLIPA14 act as negative regulators to regulate melanization (31, 43, 54). Our results provide a basis for understanding the complex regulatory network in mosquito melanization.

## Data availability statement

The mass spectrometry proteomics data presented in the study have been deposited to the ProteomeXchange Consortium via the PRIDE (56) partner repository with the dataset identifier PXD033507 and the data are also deposited into Science Data Bank (Data doi: 10.57760/sciencedb.j00001.00460, https://www.scidb.cn/s/MB7VVn).

## Ethics statement

The animal study was reviewed and approved by Animal Care and Use Committee of the Institute of Zoology, Chinese Academy of Sciences.

## Author contributions

YJ, ZZ, and YW conceived and designed the experiments. YJ and TL performed the experiments and provided critical reagents. YJ, ZZ, and YW interpreted the data and wrote the manuscript. ZZ and YW supervised the study. All authors contributed to the article and approved the submitted version.

## Funding

This work was supported by National Science Foundation of China Grant No. 32090011, 31872297, Strategic Priority Research Program of Chinese Academy of Sciences (Grant No. XDPB16).

## Acknowledgments

We thank Prof. Haobo Jiang from Oklahoma State University for critical suggestions of the manuscript. We thank LetPub (https://www.letpub.com.cn/) for its linguistic assistance during the preparation of this manuscript.

## Conflict of interest

The authors declare that the research was conducted in the absence of any commercial or financial relationships that could be construed as a potential conflict of interest.

## Publisher’s note

All claims expressed in this article are solely those of the authors and do not necessarily represent those of their affiliated organizations, or those of the publisher, the editors and the reviewers. Any product that may be evaluated in this article, or claim that may be made by its manufacturer, is not guaranteed or endorsed by the publisher.

## Abbreviations

RNAi, RNA interference; dsRNA, double-stranded RNA; AMP, antimicrobial peptide; HP, hemolymph protease; SP, serine protease; SPH, serine protease homolog; CLIP, clip domain serine protease; cSPH, CLIPs homolog; PO, phenoloxidase; PPO, prophenoloxidase; IEAR, acetyl-Ile-Glu-Ala-Arg-*p*-nitroanilide; IMP, immune melanization proteases; DOPA, dihydroxyphenylalanine; LC-MS/MS, liquid chromatography-tandem mass spectrometry.
