# Supplementary material for: Aedes aegypti CLIPB9 activates prophenoloxidase-3 in the presence of CLIPA14 after fungal infection
Source: Front Immunol. 2022 Jul 28;13:927322. doi: 10.3389/fimmu.2022.927322 (PMC9365933; doi:10.3389/fimmu.2022.927322)
Supplement: Supplementary file 1 [file DataSheet_1.pdf]

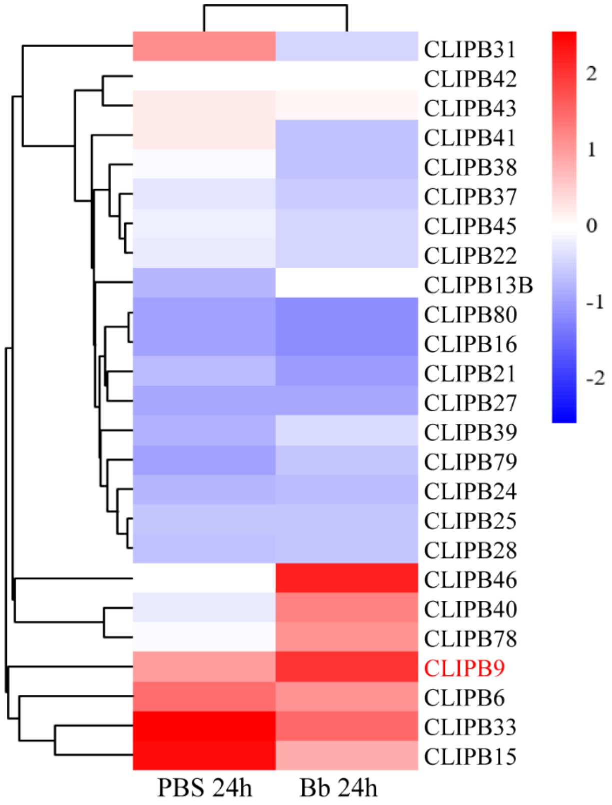

Figure S1. Hierarchical clustering of differentially expressed CLIPB subfamily proteins in mosquitoes after *B. bassiana* infection. Transcript abundance of each gene was calculated using the Hiplot website (https://hiplot.com.cn/basic/heatmap). The color scale indicates the abundance deviation from the median for each gene. The up-regulated genes were provided by previously published transcriptome data (1).

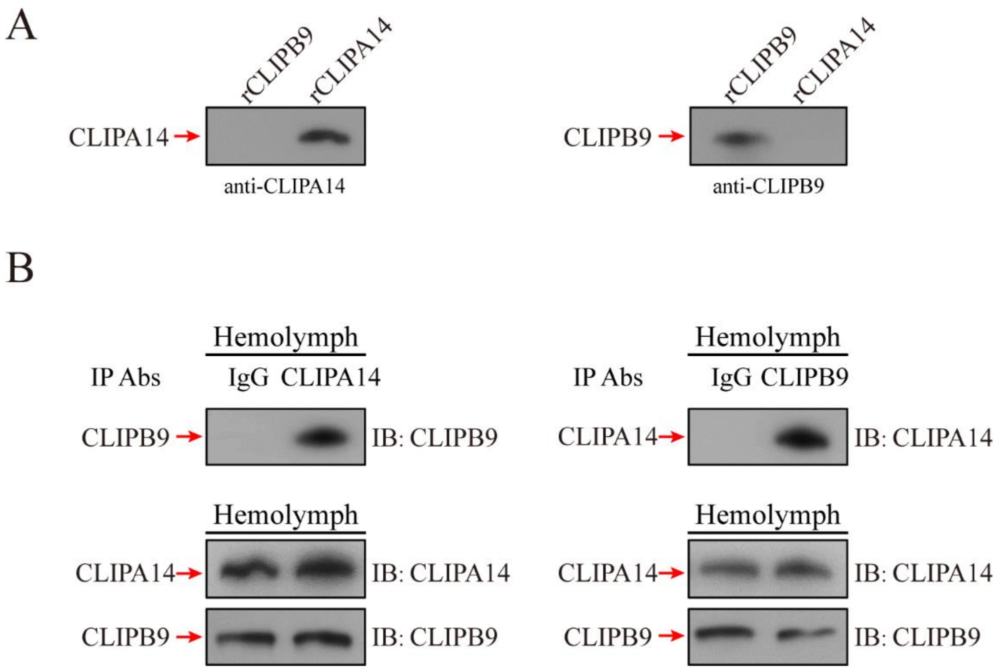

Figure S2 Confirmatory co-immunoprecipitation.

(**A**) Characterization of the specificity of CLIPB9 and CLIPA14 antibodies. **(B)** Representative Co-IP experiments revealed the interaction between CLIPB9 and CLIPA14. The immune complexes were formed by pre-incubation with anti-CLIPA14 (IP CLIPA14) and revealed with CLIPB9 antibody (left), or by pre-incubation with anti-CLIPB9 (IP CLIPB9) and revealed with CLIPA14 antibody (right). For IP-negative control, non-immune IgG were used.

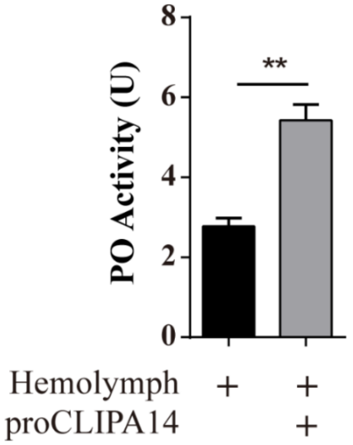

Figure S3. Characterization of the CLIPA14 protein *in vitro*.

Hemolymph samples diluted 10-fold were incubated with proCLIPA14 to detect PO activity.

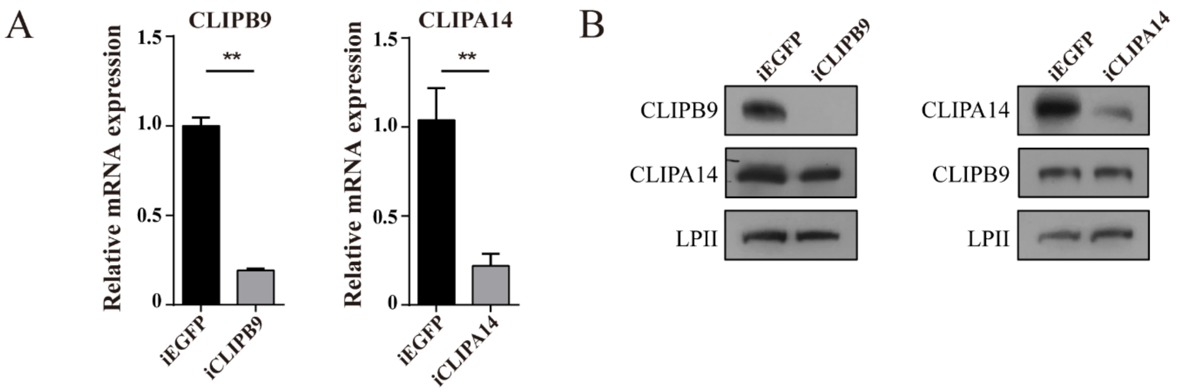

Figure S4. The RNAi efficiency of CLIPB9 and CLIPA14 were verified by qRT-PCR and immunoblotting.

(A) The RNAi efficiency of CLIPB9 and CLIPA14 were verified by qRT-PCR. The mRNA abundances of CLIPB9 and CLIPA14 were tested in relative dsRNA injected mosquitoes. **(B)** The specificity of CLIPB9 and CLIPA14 antibodies.

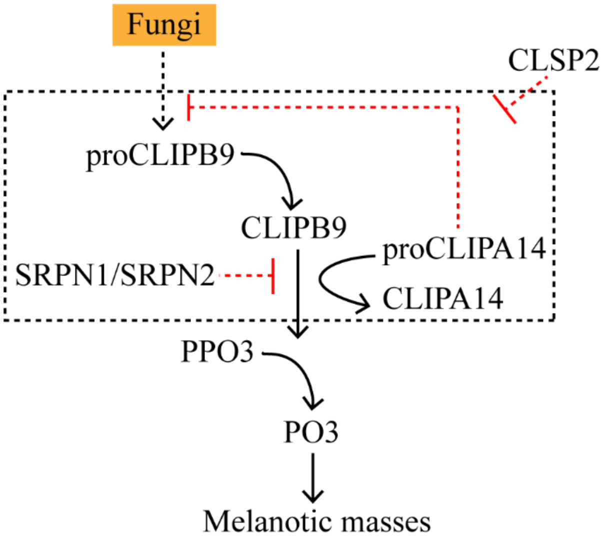

Figure S5. Activation pathway of PPO3 in *Ae. aegypti*.

After fungal infection, CLIPB9 is cleaved through a series of cascades, leading to the activation of PPO3. The cofactor CLIPA14 can enhance PO activity activated by CLIPB9.

1. Y. H. Wang, Y. Hu, L. S. Xing, H. Jiang, S. N. Hu, A. S. Raikhel and Z. Zou: A Critical Role for CLSP2 in the Modulation of Antifungal Immune Response in Mosquitoes. *PLoS Pathog*, 11(6), e1004931 (2015) doi:10.1371/journal.ppat.1004931
